# Creative, Antagonistic, and Angry? Exploring the Roots of Malevolent Creativity with a Real‐World Idea Generation Task

**DOI:** 10.1002/jocb.484

**Published:** 2020-12-12

**Authors:** Corinna M. Perchtold‐Stefan, Andreas Fink, Christian Rominger, Ilona Papousek

**Affiliations:** ^1^ University of Graz

**Keywords:** malevolent creativity, divergent thinking, antagonism, anger, behavioral test

## Abstract

Research is currently witnessing more investigations into malevolent creativity—creativity that is used to intentionally harm others. Inspired by previous methods to measure malevolent creativity, in the present study, we introduce a real‐world behavioral task designed to capture individuals’ capacity for using creativity for the purpose of attaining malevolent goals in response to everyday, provocative situations. In a sample of 105 students, we found malevolent creativity positively correlated with fluency in conventional creative ideation, as well as with self‐reported typical malevolent creativity behavior in daily life. Moreover, performance on the malevolent creativity task showed positive correlations with the maladaptive personality trait of antagonism (PID‐5) as well as individuals’ state anger at the beginning of the experiment. Further, our multiple regression analysis revealed that conventional creative ideation, antagonistic personality, and state anger all explained unique, non‐overlapping variance in the capacity for implementing malevolent creativity. As a whole, these findings suggest that different cognitive and affective factors, along with specific personality traits may each contribute to the expression of malevolent creativity in distinct ways. Future investigations striving to further decode the destructive potential of individuals toward others may benefit from this validated behavioral measurement approach to malevolent creativity.

Creativity—in the minds of laypeople and experts alike—is often venerated as the ability to produce ideas that are novel, original, and valuable from a socio‐cultural perspective. This definition implies that creativity is a constructive, positive, and highly desirable ability that benefits individuals as well as the greater good (e.g., Stein, 1953; Sternberg, 2010; but also see Baas, Roskes, Koch, Cheng, & De Dreu, 2019; Fink & Benedek, 2019). Still, it is increasingly recognized that creativity may also have darker aspects, and that the generation of novel, creative ideas can yield negative or harmful repercussions (James, Clark, & Cropanzano, 1999; McLaren, 1993; Cropley, Kaufman, & Cropley, 2008). In this regard, research investigates two darker sides of creativity: The term negative creativity is commonly used to describe creative ideas with “collateral damage”, that is, unintended harm to others (e.g., finding creative ways to avoid office work, which inevitably affects coworkers in a negative way; James et al., 1999). By comparison, the concept of malevolent creativity specifically denotes instances where creativity is purposely used to inflict material, mental, or physical harm on others (e.g., Cropley, Kaufman, & Cropley, 2008; Cropley, Kaufman, White, & Chiera, 2014). More precisely, malevolent creativity refers to the utilization of creative thinking ability for the pursuit of malevolent, violent, or destructive goals (Cropley et al., 2014; also see Runco, 2010). On a larger scale, malevolent creativity is reflected in creative terroristic acts, criminal entrepreneurship or unique strategies in war, whereas in everyday life, malevolent creativity may manifest in instances of creative deception, harassment, theft, and property destruction (e.g., Cropley, 2010; Harris, Reiter‐Palmon, & Kaufman, 2013). Recently, investigations have been concerned with personality traits and stable characteristics (e.g., Harris & Reiter‐Palmon, 2015; Jonason, Abboud, Tomé, Dummett, & Hazer, 2017; Lee & Dow, 2011), specific instances of malevolent creativity such as lying (e.g., Beaussart, Andrews, & Kaufman, 2013; Walczyk, Runco, Tripp, & Smith, 2008), or certain social contexts and cues that may facilitate malevolent creativity (e.g., Baas et al., 2019; Gutworth, Cushenbery, & Hunter, 2018).

In spite of these recent research efforts, a satisfying solution for the assessment of malevolent creativity is still lacking. Malevolent creativity has been measured and scored quite similar to the alternate uses task, in that participants were given standard creativity instructions and raters either selected ideas that suggested malevolence (Lee & Dow, 2011) or more comprehensively scored fluency, malevolence, and originality of ideas (Dumas & Strickland, 2018). As pointed out by other researchers, an obvious disadvantage of this approach, questioning its validity, is that the number of generated ideas that meet the criterion of negativity or malevolence is extremely limited (see Kapoor & Khan, 2016, 2017; Reiter‐Palmon, 2018). Other researchers explicitly asked participants to produce several creative ideas for malevolent purposes (e.g., to take revenge on someone that wronged them; introduced by Harris & Reiter‐Palmon, 2015; but also see Hao, Tang, Yang, Wang, & Runco, 2016; Hao et al., 2020); however, they only used ideas for one social scenario to quantify malevolent creativity, which again sets narrow limits to the quantity and broadness of idea generation.

In the present study, we aimed at expanding on and refining this performance‐based approach, by assessing malevolent creativity across several different hypothetical social situations. In our task, participants were confronted with unfair and provocative situations that depicted deliberately harmful behavior of another person and thus, more likely elicit malevolent creativity in daily life, as previously depicted in Harris and Reiter‐Palmon (2015), but also emphasized by Baas et al. (2019), and James et al. (1999). Further, compared to previous studies, we implemented a maximum performance approach (using the instruction to generate as many original malevolent ideas as possible) as well as time restrictions, which in our view, constitutes a better and necessary approximation to conventional creativity tests. Additionally, we adopted a more elaborate scoring method for malevolent creativity from Harris and Reiter‐Palmon (2015), which extends beyond the quantity of malevolent ideas as a whole, i.e., fluency in malevolent thinking (see Hao et al., 2016; Lee & Dow, 2011) and additionally emphasizes quality of ideas (also see e.g., Kapoor & Khan, 2017, 2020). To adequately incorporate the creativity aspect, we use a composite malevolent creativity score, including only ideas that are both, malevolent and original (Harris & Reiter‐Palmon, 2015; Harris et al., 2013).

Based on previous relevant research, we selected four constructs to look into constituting factors of this performance‐based concept of malevolent creativity: (a) conventional creative ideation in a verbal divergent thinking task (German Berlin Intelligence Structure Test; Jäger, Süß, & Beauducel, 1997), (b) self‐reported typical malevolent creativity behavior (Malevolent Creativity Behavior Scale; Hao et al., 2016), (c) maladaptive personality (Personality Inventory for the DSM‐5; American Psychological Association, 2013), and (d) the influence of participants’ current angry mood on malevolent creativity (Profile of Mood States, Dalbert, 1992).

## Linking Malevolent Creative Ideation To Conventional Creative Ideation

To date, no study specifically examined links between malevolent creativity and conventional creative ideation when both are measured in performance tests. Previously, positive correlations were found between self‐reported creative potential and self‐reported malevolent creativity behavior in real life (Hao et al., 2016, 2020). Tapping into creative performance, Jonason et al. (2017) also noted positive correlations between other‐rated creativity and incidental harmfulness of ideas generated in an alternate uses task (Jonason et al., 2017). However, Dumas and Strickland (2018) reported that while unsolicited malevolence of ideas correlated with originality of ideas in an alternate uses task, it was uncorrelated with the total number of generated ideas. Yet, since instructions in our task match those in classic creativity assessment (i.e., maximize fluency and originality of ideas in a limited amount of time), (H1) a positive correlation among indicators of malevolent creativity and conventional creative ideation was expected. Typical malevolent creativity behavior in daily life demonstrated positive correlations with fluency and originality of malevolent ideas in previous studies (Hao et al., 2016, 2020), which is why we expected (H1a) positive correlations with malevolent creativity in the present study as well.

## Linking Malevolent Creativity To Maladaptive Personality Traits

Notwithstanding considerable method variance, there is also ample evidence that malevolent creativity is linked to various aspects of aggressive behavior (e.g., Hao et al., 2016, 2020; Harris & Reiter‐Palmon, 2015; Lee & Dow, 2011). Additionally, malevolent creativity shows positive correlations with psychopathy as well as machiavellism (Jonason et al., 2017), while negative links were obtained for conscientiousness (Lee & Dow, 2011). Accordingly, we expected (H2) a positive correlation between malevolent creativity and the PID‐5 domain of antagonism in particular, since antagonistic punishment tendencies feature prominently in malevolent creativity (see Lee & Dow, 2011).

## Linking Malevolent Creativity To State Anger

We were additionally interested in the influence of participants’ current angry mood on malevolent creativity. Situational factors have been shown to affect various indicators of malevolent creativity, with recent findings suggesting that social threat (Baas et al., 2019) and certain environmental cues like goal framing (Gutworth et al., 2018) may influence the expression of malevolent creativity above and beyond individual differences in personality, even antagonism. Since hostile traits and anger are generally considered independent constructs (e.g., Eckhardt, Norlander, & Deffenbacher, 2004), both may facilitate malevolent creativity independently from each other. However, to date, the specific contribution of negative mood and affect to malevolent creativity in a performance test remains untested. We expected (H3) a positive correlation between malevolent creativity and participants’ state anger at the time of their study participation.

In addition to these expected positive correlations, we pursued the research question whether conventional creative ideation, individual differences in personality, and current affect each explain unique, non‐overlapping variance in the capacity for implementing malevolent creativity, or whether it is a combination of all three factors can best explain individuals’ malevolent creativity (RQ1).

## METHODS

### Participants

Participants were recruited online via social media, and offline via posters at several university campuses. Individuals were included in the study if they reported no neurological disease and no use of psychoactive medication. Out of 110 interested individuals, three failed to show up at the agreed appointment, and two were excluded from data analysis after testing: one due to noncompliance with test instructions, and one due to current anger ratings markedly higher than the sample mean (> five standard deviations). The final sample comprised 105 participants (58 women) aged between 18 and 39 years (*M* = 22.67, *SD* = 4.53), 87.7% of which were students enrolled in various fields. The study was approved by the authorized ethics committee. Written informed consent was obtained from all participants. The required sample size was estimated a priori with G*Power (*α* = .05, 1 – *β* = 0.80). Since effect sizes observed in previous relevant research varied markedly (*f* ^2^ = .08 to .33 for links between malevolent creativity and aggressive traits; Hao et al., 2016; Harris & Reiter‐Palmon, 2015; Lee & Dow, 2011; *f* ^2^ = .08. to .15 for links between general creative potential and malevolent creativity; Hao et al., 2020; Jonason et al., 2017), we based our calculations on *f* ^2^ = .15, which suggested a minimum of 103 participants for a multiple regression approach.

### Malevolent Creativity

**Figure 1:**
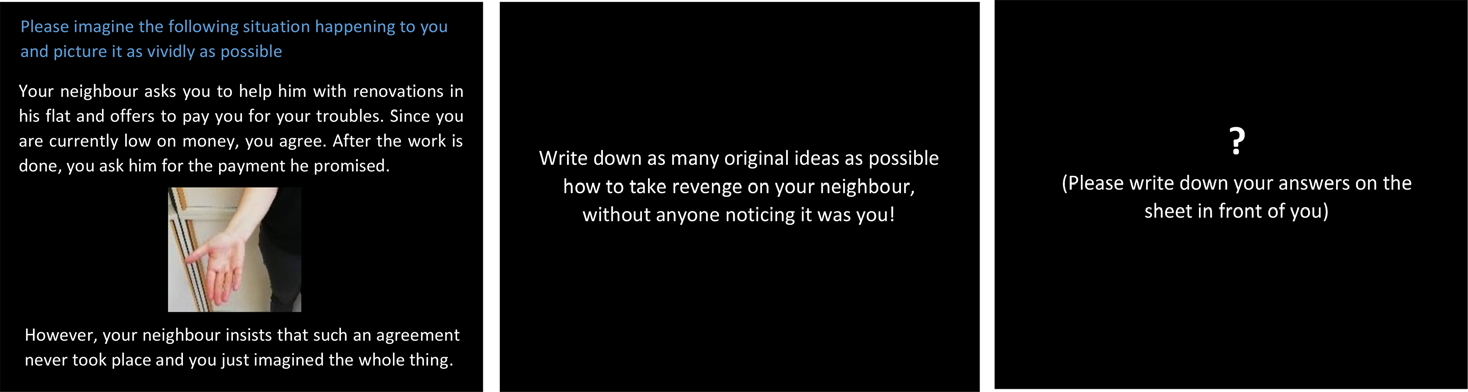
Schematic representation of the Malevolent Creativity Task (MCT). *Note*. Individuals were presented with a negative social situation and subsequently received specific instructions to take revenge/sabotage the wrongdoer. Then, they were given 3 min to generate and write down as many original ideas as possible as how to deal with the situation. The next situation appeared at the sound of a short tone via headphones.

Based on accounts that malevolent creativity is best examined in situations likely to elicit harmful behavior in daily life (e.g., unfair or provocative contexts; Baas et al., 2019; Harris & Reiter‐Palmon, 2015; James et al., 1999), the malevolent creativity task (MCT) consisted of four realistic, open‐ended problems that depicted some sort of unfair behavior from peers/associates. Each of the situations was designed to elicit at least moderate anger with the participants, yet still be relatable in terms of the plausibility to actually occur in everyday life. In the money item (a), for instance, participants face the following scenario: “Your neighbor asks you to help them with renovations in their flat and offers to pay you for your troubles. Since you are currently low on money, you agree. After the work is done, you ask them for the payment they promised. However, your neighbor insists that such an agreement never took place and you just imagined the whole thing”. In the other items, individuals are confronted with a rude classmate spilling coffee on an expensive book (b), an inconsiderate roommate throwing a party during exam season (c), and an unfair romantic rival (d). See Appendix S1 for details on all vignettes and example ideas. Participants were instructed to generate as many original ideas as possible to react to the unfair behavior depicted in these situations in order to get back at or sabotage the wrongdoer. In order to emphasize the creative aspect of the task, participants were told to try to come up with original ways of revenge without their actions tracing back to them. However, they were also informed on the hypothetical nature of the task, i.e., that they may produce ideas that are not typical for them or which they would not necessarily act out in daily life. This addendum to the instruction was made in order to counter individuals’ reluctance to openly express creative malevolent ideas due to concerns of social desirability (e.g., Hao et al., 2016). Before the beginning of the task, a practice item was given to clarify instructions. All situations were presented on a computer screen and were supplemented by a matching photograph. Participants were told to imagine the situation happening to them and to try and picture it as vividly as possible. Then, a situation‐specific instruction for idea generation followed. Subsequently, at the appearance of a white question mark on screen, participants wrote down their ideas on a sheet in front of them. Each idea generation phase lasted for 3 min, matching standard installments of creative ideation tasks that emphasize fluency as well as originality aspects of creative ideation (2–5 min per item; e.g., Beaty, Silvia, Nusbaum, Jauk, & Benedek, 2014; Benedek, Mühlmann, Jauk, & Neubauer, 2013; Fink et al., 2017; Grabner, Krenn, Fink, Arendasy, & Benedek, 2018). After the allotted time, a short tone indicated a new vignette appearing on the screen. To enable testing in groups, participants wore noise‐cancelling headphones. At the end of the paradigm, participants rated how provoked individuals would feel when confronted with the depicted situations in real life (7‐point scales ranging from 0 “not provoked at all” to 6 “very provoked”; *M* = 4.37, *SD* = 1.02, *α* = .63). In one‐sample t‐tests, provocation ratings of all four vignettes differed significantly from zero (*t*‐values from 23.66 to 35.91, all *p*‐values <.001), indicating that all depicted vignettes constituted situations in daily life that may potentially evoke malevolent creativity (e.g., Harris & Reiter‐Palmon, 2015; James et al., 1999). For a schematic representation of the task, please see Figure 1.

**Figure 2:**
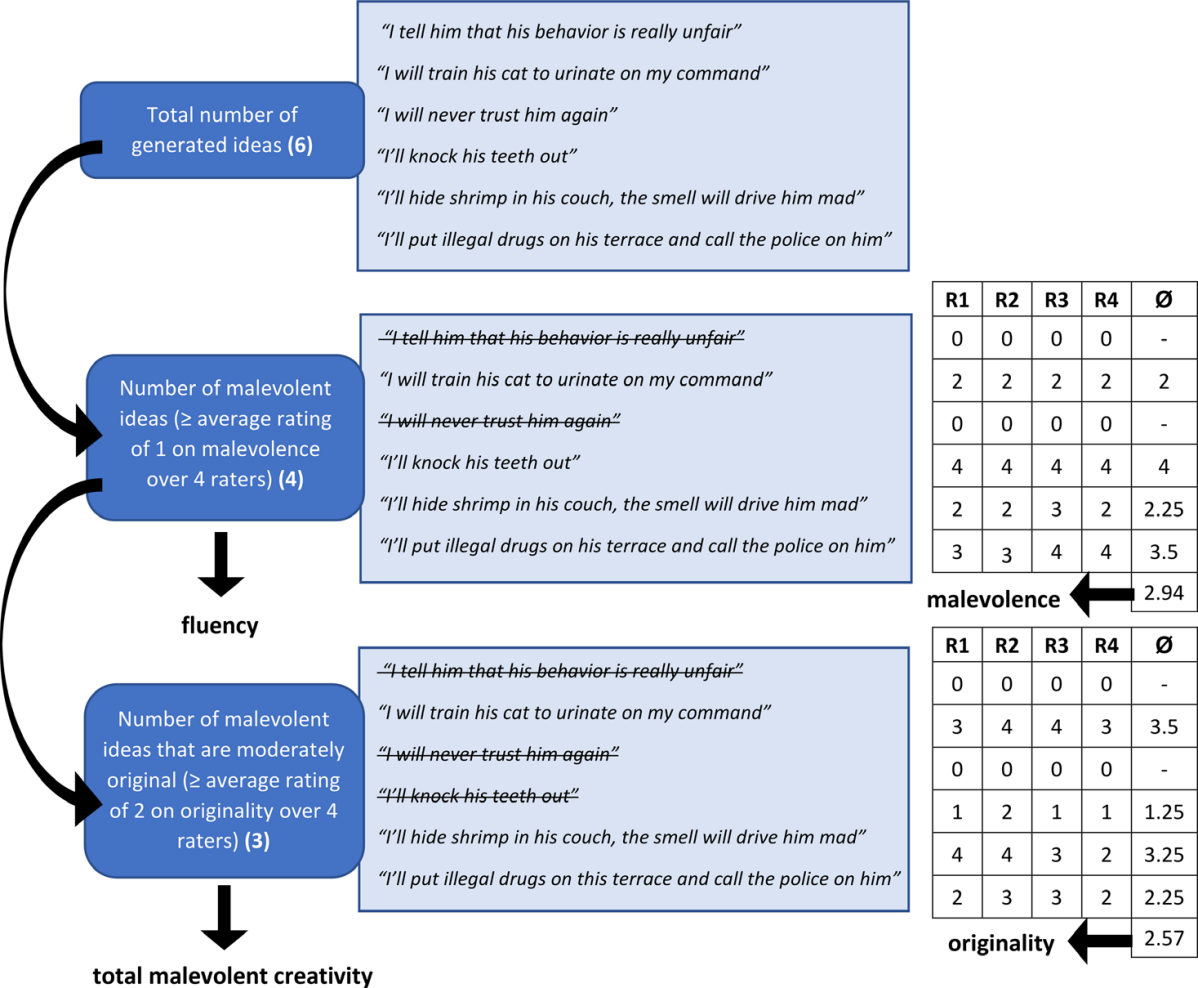
Exemplary scoring approach for the MCT item 1 (neighbour). *Note*. Of six generated ideas, four meet the criterion for malevolence and count towards MCT fluency. Of these four malevolent ideas, three meet the criterion for moderate originality and count towards total malevolent creativity in the MCT. Separate scores are calculated for malevolence (average of malevolence ratings over 4 raters) and originality (average of originality ratings over 4 raters) per participant and item.

The fluency index was calculated as the total number of non‐identical ideas generated over the four vignettes that were at least slightly malevolent or included a minor aspect of malevolence (91.2 percent of all ideas). This was determined by four experienced raters, with an ICC (two‐way, consistency) of .98. Originality was scored by having the same four raters evaluate the generated ideas for originality (cf. Consensual Assessment Technique; Amabile, 1982; see also, e.g., Perchtold et al., 2018; Rominger et al., 2019) on a 4‐point Likert scale from 1 (*not original*) to 4 (*very original*). Inter‐rater reliability was ICC = .91. Additionally, since we observed a large range of malevolence embedded in participants’ ideas (e.g., slightly malevolent: *ignoring the wrongdoer in future situations of need*; highly malevolent: *hiring local criminals to ambush the wrongdoer and beat some sense into them*), the same raters also rated the degree of malevolence on a 4‐point Likert scale, where a rating of 1 denoted slightly malevolent ideas (e.g., talking badly about the wrongdoer, making false promises, etc.) and a rating of 4 denoted highly malevolent ideas (e.g., framing the wrongdoer for a crime, bodily harm, etc.; ICC = .88). For a similar rating approach to scoring originality and valence in negative creativity, see Kapoor and Khan (2017, 2020). Details on the rating scales are reported in the Supplementary Appendix. Finally, as the primary variable of interest, a composite malevolent creativity score was computed. This conforms to the idea that “true” malevolent creativity requires ideas to qualify as both, malevolent, and original. As such, this total malevolent creativity score was computed by summing up all malevolent ideas with an average originality rating of 2 at minimum (*moderately original*). See Harris et al. (2013), and Harris and Reiter‐Palmon (2015), for a similar approach. For a schematic representation of the applied scoring approach, see Figure 2.

### Conventional Creative Ideation

The four verbal imagination subscales of the German Berlin Intelligence Structure Test (BIS; Jäger et al., 1997) require participants to produce and write down as many different ideas as possible (e.g., alternate uses, insights) in a limited amount of time (2–2.5 min). The fluency score refers to the total number of generated, non‐redundant ideas. For the subtest of alternate uses, four independent and experienced raters judged the originality of ideas on a four‐point Likert scale from 1 (*not original*) to 4 (*very original*), ICC = .76. Ratings were averaged across ideas and raters, resulting in one originality measure per participant.

### Self‐reported Typical Malevolent Creativity Behavior

The Malevolent Creativity Behavior Scale (MCBS; Hao et al., 2016) asks participants to indicate how often they engage in malevolent creativity behaviors in everyday life (e.g., deceptions, tricks, lies, revenge, etc.). It consists of 13 items rated on a 5‐point Likert scale from 0 (*never*) to 4 (*usually*; *α* = .87; e.g., “*How often do you engage in original forms of sabotage*?” or *“How often do you play tricks on people as revenge?”*).

### Current angry Mood

Participants rated three items for their current experience of anger (before the beginning of the experimental tasks) on a 7‐point Likert scale ranging from 1 (*not at all*) to 7 (*very strong*; *α* = .79, *M* = 1.36, *SD* = 0.73; Profile of Mood States, Dalbert, 1992). Participants completed the entire Profile of Mood States; however, for the purpose of the present study, only the state anger ratings were analyzed.

### DSM‐5 Personality Traits

The Personality Inventory for DSM‐5 (PID‐5, German version; Zimmermann et al., 2014) is a 220‐item questionnaire assessing personality traits according to the DSM‐5 trait model (Section III, Emerging Measures and Models, Criterion B; Krueger, Derringer, Markon, Watson, & Skodol, 2012). Items are rated on four‐point Likert scales, from 0 (*very false or often false*) to 3 (*very true or often true*). The PID‐5 consists of 25 trait facet scales, comprising five broad personality domains. These domains represent a maladaptive extension of the classic five‐factor model of personality (Suzuki, Griffin, & Samuel, 2017; Thomas et al., 2013). Domain scores were calculated by averaging the facet scores contributing primarily to the specific domain as instructed by the American Psychiatric Association (Negative Affect, *α = *.79, *M* = 0.90, *SD* = 0.57; Detachment, *α = *.83, *M* = 0.68, *SD* = 0.55; Antagonism, *α = *.76, *M* = 0.82, *SD* = 0.46; Disinhibition: *α = *.70, *M* = 0.84, *SD* = 0.45; Psychoticism, *α = *.76, *M* = 0.83, *SD* = 0.52). Scores also show adequate variability and validity in non‐clinical community and student samples with scores in the lower ranges of the scales (Bastiaens, Smits, De Hert, Vanwalleghem, & Claes, 2016; De Fruyt et al., 2013; Papousek et al., 2018).

### Procedure

**Figure 3:**
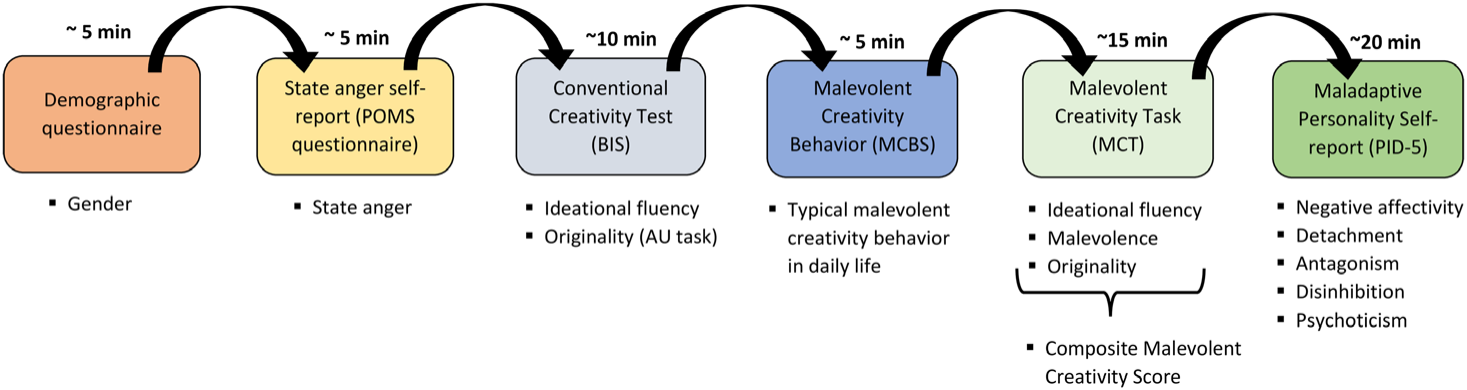
Flow diagram for the study procedure.

Participants were tested in groups of two to seven. The testing took place in an examination room that featured individual workspaces separated by partitions in order to allow for undisturbed completion of the tests and questionnaires. After the short demographical questionnaire, participants first rated their current mood, and then completed the BIS, followed by the MCBS, the MCT, and finally the PID‐5. This order of presentation was chosen to avoid carryover effects from malevolent creativity on the verbal creativity test and mood ratings and was the same for all participants. For a flow chart of the study procedure, please see Figure 3.

### Statistical Analysis

We computed Pearson correlations among all indices of malevolent creativity (fluency, originality, malevolence, total malevolent creativity) and conventional creative ideation (fluency, originality; H1), as well as among indices of malevolent creativity and self‐reported malevolent creativity behavior (H1a). Pearson correlations were also computed to examine links among malevolent creativity and maladaptive personality traits (H2), as well as state anger (H3). Next, a four‐step multiple hierarchical regression analysis was conducted in order to determine the relative significance of factors indicative of malevolent creativity (RQ1). This analysis conforms to the statistical question whether it is shared variance of several of these factors that accounts for their correlations with malevolent creativity, or whether these predictors explain unique variance in malevolent creativity, that is, variance not shared with other predictors. In the first step, gender was entered as a predictor to control for potential gender differences previously reported in literature on malevolent creativity (Dumas & Strickland, 2018; Harris & Reiter‐Palmon, 2015; Lee & Dow, 2011; however, for an interesting, more recent perspective on gender similarities in negative creativity, see Kapoor, 2019). Further, gender differences were previously reported for the PID‐5 personality trait domains (particularly Negative Affectivity and Antagonism; e.g., Bastiaens et al., 2016). At step 2, conventional verbal creativity (fluency) was entered, followed by the five PID‐5 personality trait domains at step 3. Lastly, at step 4, participants’ state anger was entered to examine whether it explained a significant amount of variance in malevolent creativity, over and above variance afforded by gender, conventional creativity, and maladaptive personality. Total malevolent creativity served as the dependent variable. Individuals’ self‐reported malevolent creativity behavior (MCBS) was not included in the model, since it was primarily assessed for examining the ecological validity of the MCT. A significance level of p < 0.05 (two‐tailed) was used.

## RESULTS

### Preliminary Analysis

Descriptive statistics and internal consistency reliabilities (Cronbach's alpha) for all creativity measures are reported in Table 1. Total malevolent creativity for each of the four situations differed slightly, with significantly lower malevolent creativity evoked by situation 4 (*M* = 1.14, *SD* = 1.17) than for the situation 3 (*M* = 1.55; *SD* = 1.40; *p* = .047), with no significant differences among all other situations (situation 1: *M* = 1.48, *SD* = 1.51; situation 2: *M* = 1.21, *SD* = 1.53). Slight differences between items also emerged for other MCT indicators, but despite being statistically significant, they were small (please see Supplementary Appendix).

**Table 1:** Descriptive Statistics for the Creativity Measures

Creativity Measure	*α*	*M*	*SD*	Min	Max
Malevolent Creativity (MCT)					
Fluency	.89	16.38	7.49	1	36
Originality	.71	1.80	0.38	1	3.13
Malevolence	.66	2.15	0.35	1	2.96
Malevolent creativity	.78	5.38	4.38	0	17
Conventional Creative Ideation (BIS)					
Fluency	.78	30.89	9.07	15	65
Originality (alternate uses)		1.83	0.28	1.25	2.5
Typical Malevolent Creativity Behavior (MCBS)					
Self‐reported malevolent creativity	.87	10.63	7.59	0	36

Note.

*α* = Cronbach's alpha between the four MCT items, the four subtests of the BIS, and the three subscales of the MCBS in this study, respectively; *M* = mean value; *SD* = standard deviation, Min = Minimum; Max = Maximum; *N* = 105.

### MCT Performance and Other Creativity Measures (H1, H1a)

There was a significant positive correlation between total malevolent creativity and ideational fluency in conventional creative ideation (*r* = .36, *p* <.001), supporting H1. This relationship was also present for MCT fluency (*r* = .28, *p* = .004), but not for MCT originality (*r* = .07, *p* = .505) and MCT malevolence (*r* = −.12, *p* = .214). All indicators of malevolent creativity (even originality) were uncorrelated with originality in the conventional verbal creativity test (all *p*’s >.281). Individuals scoring higher on fluency in the MCT also demonstrated greater malevolence (*r* = .41, *p* <.001) and originality (*r* = .23, *p* = .018) in malevolent creativity. Total malevolent creativity also positively correlated with self‐reported typical malevolent creativity behavior in real life (*r* = .34, *p* <.001), supporting H1a. See Table 2 for a summary of intercorrelations.

**Table 2:** Intercorrelations of All Creativity Measures

	MCT Total	MCT‐flu	MCT‐org	MCT‐mal	BIS‐flu	BIS‐org	MCBS
MCT‐flu	**.65**	–					
MCT‐org	**.71**	**.23**	–				
MCT‐mal	**.55**	**.41**	**.62**	–			
BIS‐flu	**.36**	**.28**	.07	−.12	–		
BIS‐org	.13	.14	.11	.11	.10	–	
MCBS	**.34**	**.25**	**.24**	**.31**	.06	**.28**	–

Note.

MCT = Malevolent Creativity Task; BIS = Berlin Intelligence Structure Test; MCBS = Malevolent Creativity Behavior Scale; flu = fluency, org = originality, mal = malevolence. Significant correlations are highlighted in bold (*p* < .05).

### MCT Performance and Maladaptive Personality Traits (H2)

There was a significant positive correlation between total malevolent creativity and antagonism (*r* = .42, *p*<.001), supporting H2. Supplementary analyses revealed that correlations with antagonism were present for all indices of malevolent creativity at least at trend level (MCT fluency: *r* = .30, *p* = .002; MCT malevolence: *r* = .20, *p* = .042; MCT originality: *r* = .18, *p* = .065). Moreover, malevolent creativity was positively correlated with Psychoticism (*r* = .22, *p* = .026). There were no significant correlations for the PID‐5 dimensions of Negative Affectivity, Detachment, and Disinhibition (all *p*’s >.270).

### MCT Performance and State Anger (H3)

There was a significant positive correlation between malevolent creativity and individuals’ angry mood at the day of testing (*r* = .30, *p* = .002), supporting H3. Supplementary analyses revealed that correlations with state anger were present for malevolence (*r* = .24, *p* = .012) and at trend level, for MCT originality (*r* = .18, *p* = .061), but not for MCT fluency (*r* = .16, *p* = .101).

### Contributions to Total Malevolent Creativity: Conventional Verbal Creativity, Maladaptive Personality Traits, and State Anger (RQ1)

In Table 3, the findings of the hierarchical multiple regression analysis are summarized. At step one, gender significantly correlated with malevolent creativity (*r* = −.19; *F*(1,103) = 4.03, *p* = .047), indicating that men showed higher malevolent creativity on the MCT than women (men: *M* = 6.32, *SD* = 4.09; women: *M* = 4.62, *SD* = 4.49). At step 2, in addition to gender, conventional creativity (fluency) significantly correlated with total malevolent creativity, explaining additional 14% of variance (*sr* = .36, *p* <.001; *F*(2,102) = 11.08, *p* <.001). At step 3, maladaptive personality traits explained additional 15% of variance of total malevolent creativity (*F*(7,97) = 5.73*, p* <.001). Only antagonism explained unique portions of variance in malevolent creativity (*sr* = .26, *p* = .002). The contributions of Negative Affectivity, Detachment, Disinhibition, and Psychoticism were non‐significant (all *p*’s >.249). Moreover, while the contribution of conventional creativity remained significant (*sr* = .35, *p* <.001), the contribution of gender became non‐significant (*sr* = −.16, *p* = .072). Entering state anger at step 4 in the model additionally increased the explained amount of variance in total malevolent creativity by 4% (*sr* = .19, *p* = .028; *F*8(96) = 5.84, *p* <.001). Both the contributions of conventional creativity and antagonism remained significant. Running a supplementary regression model for conventional creativity, antagonism, and state anger (controlling for gender) corroborated that all three variables explained unique, non‐overlapping variance in malevolent creativity, which amounted to 28% (*F*4(100) = 11.78; *p* <.001). Antagonism and state anger were not correlated (*r* = .10, *p* = .318).

**Table 3:** Summary of Hierarchical Regression Results for Total Malevolent Creativity

Dependent Variable: Total malevolent creativity	zero‐order	Step Number			
		1	2	3	4
	r (*p*)	sr (*p*)	sr (*p*)	sr (*p*)	sr (*p*)
Gender	**‐.19 (.047)**	−**.19 (.047)**	−**.22 (.015)**	−.16 (.072)	−.10 (.253)
Conventional creative ideation (fluency)	**.36 (<.001)**		**.38 (<.001)**	**.32 (<.001)**	**.32 (.001)**
Maladaptive Personality					
Negative affectivity	.05 (.622)			.10 (.249)	.05 (.539)
Detachment	.09 (.359)			−.01 (.912)	−.02 (.834)
Antagonism	**.42 (<.001)**			**.26 (.004)**	**.26 (.002)**
Disinhibition	.11 (.270)			−.02 (533)	−.05 (563)
Psychoticism	.**22 (.026)**			.05 (.533)	.05 (526)
State anger	**.30 (.002)**				**.19 (.028)**
*R* ^2^		.04	.18	.29	.33
∆*R* ^2^		**.04 (.047)**	**.14 (<.001)**	**.15 (.012)**	**.04 (.028)**

Note.

*R* ^2^ = proportions of variance explained by the model, ∆*R* ^2^ = change in *R* ^2^, *r* = zero‐order correlation, sr = semipartial correlation. Significant correlations are highlighted in bold (*p* < .05).

## DISCUSSION

The present study provided evidence of cognitive, situational, and individual factors that each contribute the generation of creative ideas intended to harm others. First, our results confirmed the expected positive association between malevolent creativity and conventional creative cognition (H1). This finding is noteworthy, since so far, previous studies only linked creativity in standard tests (alternate uses) to malevolence of answers in the same test (e.g., Dumas & Strickland, 2018) or to self‐reported likelihood for positive and negative creative behavior (Kapoor & Khan, 2016). This relationship seemed to be predominantly driven by ideational fluency in both tasks, which suggests that malevolent creativity may, in part, emerge from similar cognitive processes that allow for fluent generation of ideas. In contrast to the positive correlations of fluency, originality scores were not related. This may suggest that high originality of malevolent ideas do not necessarily arise from a greater general potential to retrieve more uncommon, unique and thus, deviant associations.

Malevolent creativity performance on the MCT was also positively correlated with self‐reported typical malevolent creativity behavior in daily life (H1a). This result corroborates and extends previous findings from a single item social revenge task (Hao et al., 2016, 2020). In our opinion, this finding underlines the practical value of soliciting malevolent creativity with explicit instructions in experimental settings, since the potential for malevolent creativity, in part, may translate to actual malevolent behavior, if individuals are pursuing malicious interpersonal goals in response to provocative situations (for criticism with this solicited approach, see Dumas & Strickland, 2018).

Greater capacity for using creativity for malevolent purposes was also associated with higher expressions of antagonism (H2), which captures aspects of manipulativeness, deceitfulness, and grandiosity, and represents the maladaptive extension of low levels of the classic “Big Five” trait agreeableness (e.g., Suzuki et al., 2017). Disagreeableness in terms of aggressive and hostile traits has been quite robustly linked to malevolent creativity in various tests (Harris & Reiter‐Palmon, 2015; Lee & Dow, 2011). It was previously contended that the ability to manipulate and deceive requires breaking with established rules and “thinking outside the box” (e.g., Runco, 2010). In support, unethical behavior, particularly dishonesty, has been found to both enhance and be enhanced by creativity (e.g., Beaussart et al., 2013; Gino & Wiltermuth, 2014). Accordingly, in the present study, higher antagonism was not only linked to higher malevolence, but also to higher ideational fluency and, at trend level, higher originality in the MCT. These correlations suggest that the relationship between antagonism and malevolent creativity is not a purely motivational one, in that antagonistic individuals are simply more reactive to provocation (e.g., Bettencourt, Talley, Benjamin, & Valentine, 2006) or less inhibited in writing down harmful ideas (e.g., Harris & Reiter‐Palmon, 2015). If this were the case, correlations with maladaptive personality likely may have been restricted to the index of malevolence alone (see Jonason et al., 2017).

Individuals’ malevolent creativity also appeared to be influenced by situational factors, in that participants reporting higher state anger at the beginning of the experiment achieved higher scores in the MCT (H3). Hao et al. (2020) recently found that approach motivation seems to promote individuals’ malevolent creativity, arguing that it may increase risk‐taking and the willingness to violate social norms (also see Friedman & Förster, 2002). Considering that anger is robustly associated with approach motivation (e.g., Carver & Harmon‐Jones, 2009; Harmon‐Jones, 2003), the results of the present study may reflect a similar effect. The correlation between state anger and malevolent (but not conventional) creativity also fits the idea that if an individuals’ mood is congruent with the framing of a certain task, a greater investment of their time and energy may enhance creative performance (e.g., De Dreu, Baas, & Nijstad, 2012).

As perhaps the most crucial finding of the present study, conventional creativity, antagonistic personality, and state anger all explained unique, non‐overlapping variance of MCT performance (RQ1). This finding underlines that cognitive and affective factors, as well as specific individual differences each contribute to the expression of malevolent creativity in unique but comparatively important ways.

Taken together, individuals scoring higher in conventional creativity tests may also achieve higher scores in tests for malevolent creativity, because they can apply their general divergent thinking faculties across different contexts. This may indicate a cognitive route to malevolent creativity. However, this overlap in cognitive processes between conventional and malevolent creativity is not nearly as large as correlations of conventional creativity with other real‐life types of creativity, e.g., creativity in generating cognitive reappraisals for negative emotional events (correlations of up to *r* = .61; Fink et al., 2017; Weber, Assuncao, Martin, Westmeyer, & Geisler, 2014). Thus, other factors must have a vital additional role. Individual differences in maladaptive personality seem to highlight a second, independent route to malevolent creativity, indicating that individuals with higher levels of antagonism are more capable of using creativity for malevolent purposes. Lastly, the unique contribution of state anger emphasizes the influence of situational affective factors in the generation of malevolently creative ideas, thus highlighting the need to incorporate measures of mood and affect in order to fully understand malevolent creativity (see Baas et al., 2019).

Among the most important strengths of the present study is the validation of a behavioral test for malevolent creativity that integrates and expands several crucial aspects of malevolent creativity assessment respectively proposed by different previous studies: (a) the assessment of malevolent creativity in ecologically valid, negative social situations that likely evoke malevolent creativity in real life (see Baas et al., 2019; Harris & Reiter‐Palmon, 2015), (b) explicitly asking participants to generate as many creative ideas as possible for malevolent purposes in terms of a maximum performance approach (see Hao et al., 2016, 2020; Harris & Reiter‐Palmon, 2015), (c) emphasizing quality aspects of malevolent creative ideas in terms of malevolence/valence and originality/uniqueness (see Kapoor & Khan, 2017, 2020), but also (d) composing a more “authentic” malevolent creativity score that incorporates both malevolence and originality of ideas (see Harris & Reiter‐Palmon, 2015; Harris et al., 2013), and (e) expanding the performance‐based approach to malevolent creativity by adopting time‐sensitive, multi‐item protocols of standard psychometric creativity tests. It is the combination and expansion of the best of previous assessment suggestions for malevolent creativity into a behavioral test that we consider a novelty of the present study, together with a more profound understanding of cognitive, situational, and individual factors that each contribute the generation of creative ideas intended to harm others.

A few limitations must be noted. The multiple regression analysis had a rather low n:k ratio (13:1), which raises potential concerns about statistical power and stability of the regression coefficients. Thus, replication of the obtained relationships in larger samples is warranted. Second, the generalization of findings requires caution, since the majority of our sample was comprised of young students. Accordingly, the findings should be replicated in other populations, for which the thematic focus of the vignettes can be easily broadened and adapted. Here, situations that provoke malevolent creativity in the workplace could be of particular interest (see James et al., 1999). Next, the cross‐sectional nature of the present study precludes any inference of causality. While it may be intuitive that maladaptive personality serves as an antecedent of malevolent creativity and previous research contends that context manipulations influence the expression of malevolent creativity (Baas et al., 2019; Gutworth et al., 2018), reciprocal mechanisms are possible and need investigating. Moreover, originality could only be scored for the subtest of alternate uses in the conventional creativity test, where instructions maximize fluency and diversity rather than originality of ideas (Jäger et al., 1997). In order to more meaningfully link originality in conventional and malevolent creativity, future studies with the MCT should employ several items of the alternate uses task with an emphasis on originality of ideas. Additionally, in order to increase differentiability and further examine the robustness of our results, the use of more fine‐grained rating scales for originality and malevolence of ideas generated in the MCT should be considered (e.g., five to six points; see Harris & Reiter‐Palmon, 2015; Kapoor & Khan, 2017).

As a final note, the newly developed malevolent creativity task used in this study showed satisfactory reliability, the range of scores indicated sufficient variance for a meaningful analysis of individual differences in the general population, and the observed relationships gave indications of the validity of the derived scores. Thus, the elaborated behavioral approach to malevolent creativity may benefit future investigations, given that the destructive consequences of malevolent creativity clearly necessitate more research into this phenomenon.

## Supporting information


**Appendix S1.** Malevolent Creativity Task (MCT).

## Data Availability

The data that support the findings of this study are available from the corresponding author upon reasonable request.
