# Supplementary material for: Creative, Antagonistic, and Angry? Exploring the Roots of Malevolent Creativity with a Real‐World Idea Generation Task
Source: J Creat Behav. 2020 Dec 12;55(3):710–22. doi: 10.1002/jocb.484 (PMC8518065; doi:10.1002/jocb.484)
Supplement: Supplementary file 1 — Appendix S1. Malevolent Creativity Task (MCT). [file JOCB-55-710-s001.docx]

**Appendix**

**Malevolent Creativity Task (MCT)**

**Situation 1**

Your neighbour asks you to help them with renovations in their flat and offers to pay you for your troubles. Since you are currently low on money, you agree. After the work is done, you ask them for the payment they promised. However, your neighbour insists that such an agreement never took place and you just imagined the whole thing. You decide not to take this.

Example answer:

*“I will post my neighbors phone number online with the title “Free Computer, please only call at night“*

*“I will release countless cockroaches into their flat”*

**Situation 2**

You have saved up for a while to be finally able to buy yourself a very expensive science book. Just as you are about to enter the lecture hall at the university, a colleague of yours bump into you hard and spills all her coffee on your new book. Instead of apologizing, that colleague berates you to pay more attention next time and marches on, leaving you and your book completely drenched. You want to teach that colleague a lesson.

Example answers:

*“At the next chance, I will empty an entire cup of coffee into their purse when they aren’t looking”*

*“I will make a scene in front of the class, so everyone knows what a horrible person they are and they lose their reputation”*

**Situation 3**

Since you need to study for an important exam on the weekend, you asked your roommate for some quiet time in our shared flat. Although they have disappointed you quite often in the past, you hope that this time, they will keep their promise not to invite other people. Before the weekend, just by chance, you hear from one of your acquaintances that your roommate has announced a giant party in your flat. You have had enough and want to teach your roommate a lesson.

Example answers:

*“I will class my roommates’ parents and tell them their son has been using drugs and needs help immediately”*

*“On the day of the party, I will slip my roommate a laxative; see if they can have the party then”*

**Situation 4**

You have been flirting with a person in your circle of friends for a while now. When you want to finally ask them on a date, you find out that they already have plans with someone else. This other person has a very bad reputation and already ruined one of our relationships in the past. You want to keep this person from interfering with your relationships again.

Example answers:

“*I will spread the rumor that this person has an STD in his [crush] circle of friends*”

“*I will hack that persons’ Facebook profile and send insane-sounding messages to my crush”*

**4-point rating scale for originality of ideas generated in the MCT (item neighbour)**

**1** = not original, idea would occur to anybody

*Example: next time the wrongdoer asks for help, retaliate with the same behavior*

**2** = moderately original, idea would occur to most people in time

*Example: putting something off-putting in their mailbox (slime, litter)*

**3** = original = idea would not occur to anybody

*Example: subscribing the wrongdoer to several self-help magazines as how to become a better person*

**4** = very original, idea would only occur to very few individuals

*Example: training the wrongdoer’s cat to urinate on command*

**4-point rating scale for malevolence of ideas generated in the MCT (item neighbour)**

**1** = slightly malevolent, causing very little damage to the recipient

*Example: Talking badly about the wrongdoer, manipulating people to ignore the wrongdoer*

**2** = moderately malevolent, causing moderate damage to the recipient

*Example: playing pranks (egging their car), impersonating the wrongdoer*

**3** = malevolent, causing significant damage to the recipient

*Example: constant harassment of the wrongdoer, stealing and damaging property of the wrongdoer*

**4** = highly malevolent, causing immense to maximum damage to the recipient

*Example: poisoning the wrongdoer, framing the wrongdoer for a capital crime*

**Supplementary Analysis**

|  | Situation 1 | | Situation 2 | | Situation 3 | | Situation 4 | | p |
| --- | --- | --- | --- | --- | --- | --- | --- | --- | --- |
|  | M | SD | M | SD | M | SD | M | SD |  |
| MCT  Fluency | 4.02 | 2.24 | 3.88 | 2.17 | 4.82 | 2.27 | 3.67 | 1.97 | S1 vs S2 = 1.00  S2 vs S3 <.001  S3 vs S4 <.001  S1 vs S4 = .279  S2 vs S4 = .970 |
| MCT Malevolence | 2.39 | 0.43 | 2.25 | 0.43 | 2.27 | 0.35 | 1.90 | 0.60 | S1 vs S2 = .032  S2 vs S3 = 1.00  S3 vs S4 <.001  S1 vs S4 <.001  S2 vs S4 <.001 |
| MCT Originality | 1.99 | 0.47 | 1.73 | 0.57 | 1.86 | 0.41 | 1.81 | 0.49 | S1 vs S2 <.001  S2 vs S3 = .153  S3 vs S4 = 1.00  S1 vs S4 = .005  S2 vs S4 = .615 |

*Note.* Repeated-measures Anova, Bonferroni corrected, N = 105.
